# Optoelectronic Response to the Fluor Ion Bond on 4-(4,4,5,5-Tetramethyl-1,3,2-dioxoborolan-2-yl)benzaldehyde

**DOI:** 10.3390/ijms25095000

**Published:** 2024-05-03

**Authors:** Ulises J. Guevara, Jesús Núñez, Laura M. Pérez, Anton Tiutiunnyk, Neudo Urdaneta, Eduardo Cisternas, David Laroze

**Affiliations:** 1 Instituto de Alta Investigación, Universidad de Tarapacá, Arica 1000000, Chile; uguevara@academicos.uta.cl (U.J.G.);; 2 Departamento de Biología, Universidad Politécnica Territorial del Oeste de Sucre “Clodosbaldo Russian”, Cumaná 6101, Venezuela; 3 Departamento de Ingeniería Industrial y de Sistemas, Universidad de Tarapacá, Arica 1000000, Chile; 4 Departamento de Física, FACI, Universidad de Tarapacá, Arica 1000000, Chile; 5 Departamento de Química, Universidad Simón Bolívar (USB), Caracas 1020-A, Venezuela; 6 Departamento de Ciencias Físicas, Universidad de La Frontera, Casilla 54-D, Temuco 4811230, Chile; eduardo.cisternas@ufrontera.cl

**Keywords:** density functional theory, nuclear magnetic resonance, UV-vis, infrared spectroscopy

## Abstract

Boronate esters are a class of compounds containing a boron atom bonded to two oxygen atoms in an ester group, often being used as precursors in the synthesis of other materials. The characterization of the structure and properties of esters is usually carried out by UV-visible, infrared, and nuclear magnetic resonance (NMR) spectroscopic techniques. With the aim to better understand our experimental data, in this article, the density functional theory (DFT) is used to analyze the UV-visible and infrared spectra, as well as the isotropic shielding and chemical shifts of the hydrogen atoms ^1^H, carbon ^13^C and boron ^11^B in the compound 4-(4,4,5,5-tetramethyl-1,3,2-dioxoborolan-2-yl)benzaldehyde. Furthermore, this study considers the change in its electronic and spectroscopic properties of this particular ester, when its boron atom is coordinated with a fluoride anion. The calculations were carried out using the LSDA and B3LYP functionals in Gaussian-16, and PBE in CASTEP. The results show that the B3LYP functional gives the best approximation to the experimental data. The formation of a coordinated covalent B–F bond highlights the remarkable sensitivity of the NMR chemical shifts of carbon, oxygen, and boron atoms and their surroundings. Furthermore, this bond also highlights the changes in the electron transitions bands *n* → π* and *π* → π* during the absorption and emission of a photon in the UV-vis, and in the stretching bands of the C=C bonds, and bending of BO_2_ in the infrared spectrum. This study not only contributes to the understanding of the properties of boronate esters but also provides important information on the interactions and responses optoelectronic of the compound when is bonded to a fluorine atom.

## 1. Introduction

Materials science and engineering have evolved dramatically from empirical trial-and-error approaches, optimizations, and the use of metaheuristic methods, which are characterized by their development with the use of available technological tools. The design of new materials has become a challenge to create models by applying quantum chemistry to characterize and predict new properties [1,2]. Likewise, the deeper the knowledge of a subject, the more questions arise and with them new research challenges and opportunities. This in turn leads to the discovery of new materials and methods, and their application in various areas of science, such as chemistry, physics, and engineering, among others. In this sense, each problem solved is an opportunity for the discovery of new knowledge and advances in materials science [3,4,5,6].

A common application of these models and techniques is the identification of new compounds containing the boronate ester group. Determining the relationship between the atoms and peaks found in the spectra can sometimes be a challenging task [7,8,9,10]. The carbonyl groups (–RCOR’) of aldehydes or ketones are important in organic and biochemical syntheses, since, through an aldol condensation, they can form carbon-carbon bonds. According to some conditions of synthesis of organic compounds, in the boronic acid group (–B(OH)_2_) the breaking of the B–C bond takes place, so sometimes it is necessary to protect the group with a diol to form the boronate ester and obtain the desired product [11,12,13,14,15].

Compounds containing boronate ester groups are widely used in numerous syntheses, most notably in the Suzuki–Miyaura coupling reaction [16]. This reaction allows the union of two organic molecules to form a more complex compound that is used in the synthesis of pharmaceuticals, advanced materials, and natural products of biological interest [17]. They are useful for the detection of carbohydrates [18] and fluoride ions [19], due to their capacity to produce cyclic esters [20], since the boron atom is a Lewis acid because it has *p* orbitals available to form stable bonds. In addition, these compounds show biological activities, such as antidepressant, antialergic, anaesthetic, and anti-Alzheimer agents, as well as proteasome and lipoglycan inhibitors [21].

In solution, boronic acids and their derivatives can bind directly to compounds containing hydroxyl groups, such as glucose or glycoproteins, with a direct change in their electrochemical response [22]. These compounds have served as a useful tool for the design of new drugs. The US Food and Drug Administration (FDA) approved the use of the drug Velcade (its active agent Boterzomid contains boronic acid function) in 2003 for the treatment of adult patients with multiple myeloma (a type of bone marrow cancer), so the interest in the synthesis of new bioactive boronic acids has grown dramatically in recent years [23].

Recently, the use of first-principles calculations to complement the interpretation and assignment of chemical shifts in nuclear magnetic resonance (NMR) spectra has become very prevalent. These calculations can provide a crucial link between the structural and stereochemical details of an organic compound, with their corresponding NMR empirical chemical shifts [24,25]. For molecules of moderate to large size, the DFT method offers an option of low computational cost and good enough approximation to predict the chemical shifts of an organic compound, with the implementation of a series of exchange-correlation functionals and the inclusion of the effect of the solvent [26,27]. In this context, it is essential to know the properties and possible use of emerging compounds, so that they can be of benefit for different applications. It should be noted that recent studies have implemented a machine learning algorithm to predict ^11^B NMR chemical shifts in emerging organic compounds, adding techniques and methodology for spectral studies [28]. In this sense, it is important to have a detailed knowledge of the physical and chemical characteristics of the molecule, as well as its electronic properties. The rigorous and systematic study of a molecular structure is therefore a substantial task for the advancement of materials science.

**Figure 1:**
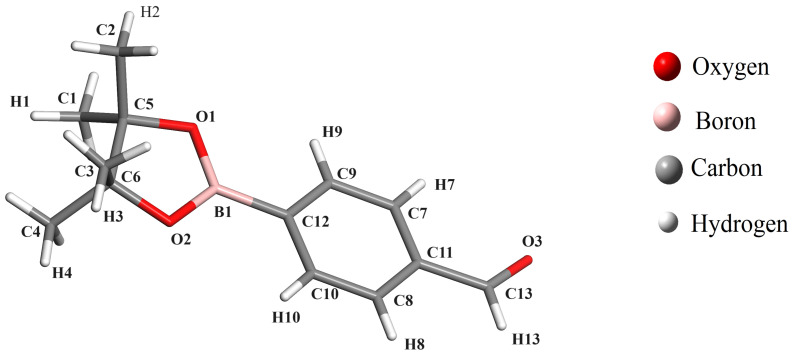
Structure of 4-(4,4,5,5-tetramethyl-1,3,2-dioxoborolan-2-yl)benzaldehyde compound (ABP).

**Figure 2:**
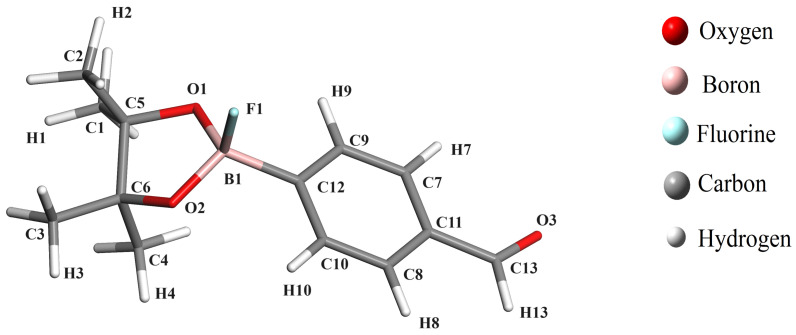
Structure of the compound 4-(4,4,5,5-tetramethyl-1,3,2-dioxoborolan-2-yl)benzaldehyde bonded with a fluorine atom (ABPF).

Boronate esters are compounds containing a boron atom bonded to two oxygen atoms in an ester group. These compounds are commonly used as starting materials for the synthesis of other organic compounds. The structure and properties of boronate esters can be effectively analyzed and characterized using infrared (IR), ultraviolet-visible (UV-vis), and NMR spectroscopies. Research in the field of crystallography and spectroscopy of these compounds is still scarce, and only a few crystal structures have been reported so far. There is ample scope for exploring new compounds and their crystallographic study, which could produce important findings in chemistry and industry. The main objective of this work is based on the study of the isotropic NMR shielding of ^1^H, ^13^C, ^11^B and ^17^O, UV-vis and IR spectral signals of 4-(4,4,5,5-tetramethyl-1,3,2-dioxoborolan-2-yl)benzaldehyde (C_13_H_17_BO_3_ hereafter ABP) and the changes of these spectroscopic properties when the ABP compound is covalently coordinated with a fluoride anion (where the ABPF anion is obtained by mixing tetrabutyl ammonium fluoride with a solution of the ABP compound in chloroform, by applying the density functional theory. The CASTEP [29] and Gaussian-16 [30] packages were used to investigate the NMR spectra of ^1^H, ^13^C, ^11^B, ^17^O, UV-Vis and IR spectra for the ABP compound (Figure 1), and the changes in these when the fluoride anion is covalently bonded with its boron atom ([C_13_H_17_BFO_3_]^−^ hereafter ABPF, see Figure 2), by of the different exchange-correlation functionals, which we highlight PBE and B3LYP. The results showed that the chemical shielding of carbon, boron, and oxygen atoms, some signals of the UV-vis and IR spectra of the studied compounds, are sensitive to the electrical environment of the boron atom. The article is organized as follows: In Section 2, the methodology is presented, while in Section 3, the results are presented and analyzed, and, finally, the conclusion is given in Section 4.

## 2. Materials and Methods

### 2.1. Synthesis and Characterization of the Compounds ABP and ABPF

Compound ABP was synthesized from the mixture of (4-formylphenyl)boronic acid (Sigma-Aldrich, Co., 2nd St 3306, St. Louis, MO 63118, USA) and 2,3-dimethyl-2,3-butanediol in diethyl ether at room temperature for 5 h [20]. The ^1^H NMR spectrum of the ABP compound dissolved in CD_3_Cl was measured on a 500 MHz spectrometer Bruker (Manning Park, MA 01821 3991 Billerica, MA, USA), model Aspect 3000. The infrared spectrum of the ABP compound was measured from a pellet consisting of a mixture of the ABP compound and KBr. A Bruker Optik GMBH Tensor 27 FT-IR spectrometer was used with Opus/IR software (Version 5), which uses the Fourier Transform method for data processing of the IR-TF spectrum.

Solutions of the compounds ABP and ABPF at 10^−5^ M and 10^−4^ M in chloroform were prepared to measure the absorption and fluorescence spectra, respectively. A standard solution of 0.1 M fluorescein sodium in NaOH aqueous solution was prepared. The UV-vis absorption spectra were measured in a Varian spectrophotometer (model CARY50) and the fluorescence spectra were measured in a Varian CARY Eclipse spectrofluorometer. The quantum yield $\Phi_{F(X)}$ were calculated from Equation (1), with respect to the fluorescein sodium obtained commercially from Sigma-Aldrich, Co. [31,32].

(1) $$\Phi_{F(X)}=\frac{A_{s}}{A_{x}}\frac{F_{x}}{F_{s}}\frac{\eta_{x}^{2}}{\eta_{s}^{2}}\Phi_{F(S)}$$

where *A* is the absorbance value at the excitation wavelength, *F* the area under the fluorescence spectrum curve, η the refractive index of the solvent, *S* refers to the standard used (in this case fluorescein sodium) and *X* the sample to be studied.

### 2.2. Computational Calculations

The different computational implementations of the Density Functional Theory (DFT) introduce several approximations, which mainly explain the deviations between theoretical results and experimental data. However, one critical approximation corresponds to the exchange-correlation functional, for which considered several cases implemented in software CASTEP (Version 20.11) and Gaussian (Version 16).

#### 2.2.1. Optimization of the Structures of BF_3_·OEt_2_, Si(CH_3_)_4_, H_2_O, ABP and ABPF in CASTEP

**Figure 3:**
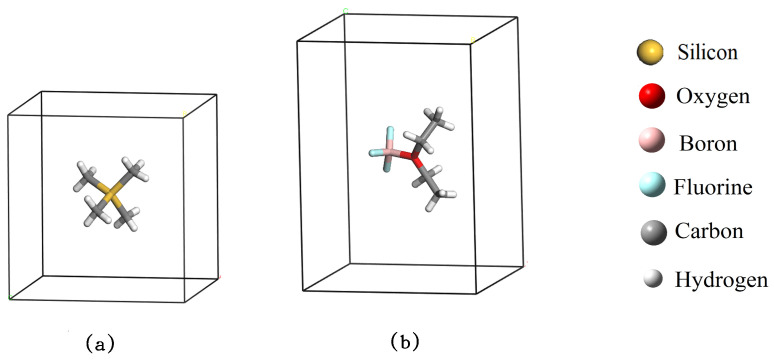
Reference compounds Si(CH_3_)_4_ (**a**) and BF_3_·OEt_2_ (**b**).

CASTEP performs calculations on periodic structures, where the atoms in one cell interact with the atoms in the neighboring cell. So, to work a molecule in CASTEP, it must be placed inside a box large enough to prevent the molecule from interacting with its neighbors at periodicity. So, a cell of 10 Å × 10 Å × 10 Å for the Si(CH_3_)_4_ molecule (Figure 3a), another cell of 10 Å × 10 Å × 15 Å is constructed for the molecule BF_3_·OEt_2_ (Figure 3b). Furthermore, cells of 12 Å ×12 Å × 20 Å were defined for the ABP compound and ABPF anion. Such selection ensures that the structures are isolated from each other.

For the optimization of the Si(CH_3_)_4_, BF_3_·OEt_2_, ABP and ABPF in the cells, Perdew–Burke–Ernzerhof exchange and correlation functionals (PBE) [33], with generalized gradient approximation and a plane wave basis set, were used. The Γ point in reciprocal space with a cutoff energy of 300.00 eV, a convergence tolerance parameter of $1.00\times 10^{-5}$ eV/atom, a maximum force of 0.05 eV/Å, a maximum stress of 0.01 GPa, and a maximum displacement of $2.00\times 10^{-4}$ Å. The pseudopotential is one generated during runtime on the server “on the fly”, i.e., a pseudopotential generated dynamically during electronic calculations instead of one calculated in advance. This brings great efficiency and flexibility to the calculations, especially in the case of large or complex systems. This approach differs from the norm-conserving pseudopotentials of the standard [34] and ultra-soft [35], in that they use tabulated data for the projections. The BFGS algorithm was used in the optimization. The results of applying this functional can be seen in the Table 1, Table 2, Table 3 and Table 4 of Section 3.1.

#### 2.2.2. Optimization of the Structures of BF_3_·OEt_2_, Si(CH_3_)_4_, H_2_O, ABP and ABPF in Gaussian-16

The Gaussian-16 software [30] works directly on the molecules, so for the optimization we worked with a basis set 6-311 + G(2d,p) [36,37,38] and the exchange-correlation functional B3LYP [39,40]. The default algorithm for the minimization is Berny’s algorithm using GEDIIS [41]. Convergence is tested against the criteria for the maximum force component $4.50\times 10^{-4}$ Ha/bohr, mean square force $3.00\times 10^{-4}$ Ha/bohr maximum step component $1.80\times 10^{-3}$ bohr, and mean square ratio step $1.20\times 10^{-3}$ bohr. The step is the change between the most recent point and the next point to be calculated (the sum of the linear and quadratic steps) [42]. The results of applying this functional can be seen the Table 1, Table 2, Table 3 and Table 4 of the Section 3.1.

#### 2.2.3. NMR of Si(CH_3_)_4_, BF_3_·OEt_2_, H_2_O, ABP and ABPF

To calculate the isotropic shielding and the chemical shift of ^1^H, ^13^C, ^17^O and ^11^B in the ABP and ABPF structures, it was necessary to optimize them and the reference compounds Si(CH_3_)_4_, H_2_O and BF_3_·OEt_2_. In the case of the shielding of ^1^H and ^13^C the reference composite Si(CH_3_)_4_ was used, for ^17^O the H_2_O molecule was used as reference and for the shielding of ^11^B, the reference composite was BF_3_·OEt_2_. Once the Si(CH_3_)_4_, BF_3_·OEt_2_, H_2_O, ABP and ABPF cells in CASTEP are relaxed, the same augmented wave pseudopotential used in the relaxation was used for the nuclear magnetic resonance calculation, with a cutoff energy of 450.00 eV. The Brillouin zone integration was defined as a 4×4×4, whereas the PBE–GGA functional was considered with a self-consistent field tolerance (SCF) of $2.00\times 10^{-5}$ eV/atom and a maximum self-consistent cycle of 100 cycles.

**Figure 4:**
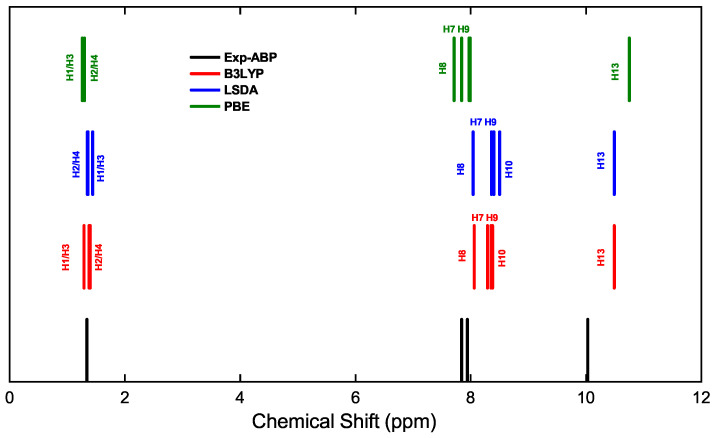
Hydrogen chemical shift for the ABP compound. Experimental NMR values (black line) are compared with different computed results using PBE with DFT + D in CASTEP (green line), LSDA in Gaussian-16 (blue line), and B3LYP in Gaussian-16 (red line).

**Figure 5:**
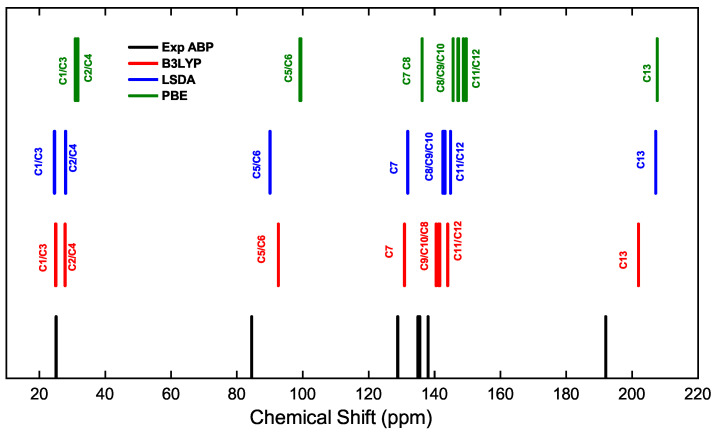
Carbon chemical shift for the APB compound. Experimental NMR values (black line) are compared with different computed results using PBE with DFT + D in CASTEP (green line), LSDA in Gaussian-16 (blue line), and B3LYP in Gaussian-16 (red line).

**Figure 6:**
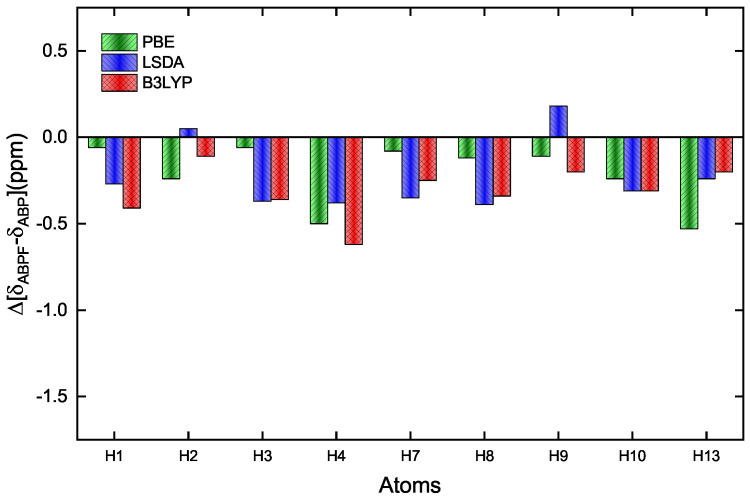
Difference of the theoretical values for the chemical shifts of H in the ABPF and ABP. The bars indicate the different functionals applied, red for B3LYP and blue for LSDA in Gaussian-16, and green for PBE in CASTEP.

**Figure 7:**
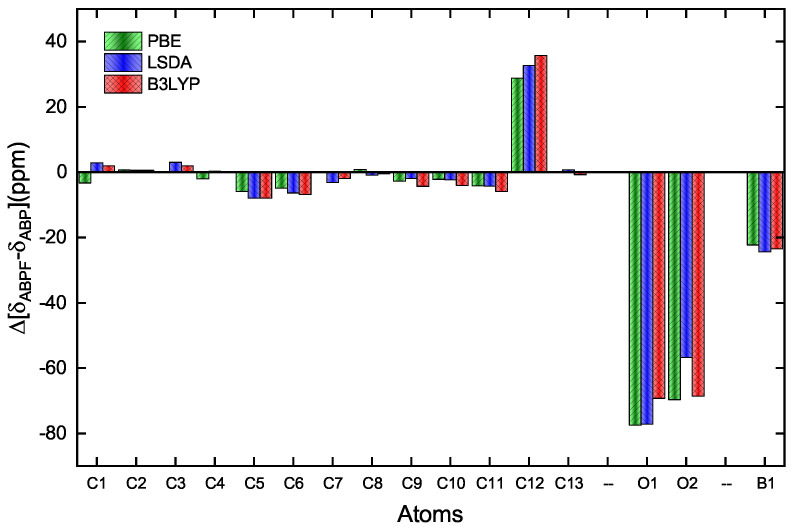
Difference of the theoretical values for the chemical shifts of C, O and B in the ABPF and ABP. The bars indicate the different functionals applied, red for B3LYP and blue for LSDA in Gaussian-16, and green for PBE in CASTEP.

**Figure 8:**
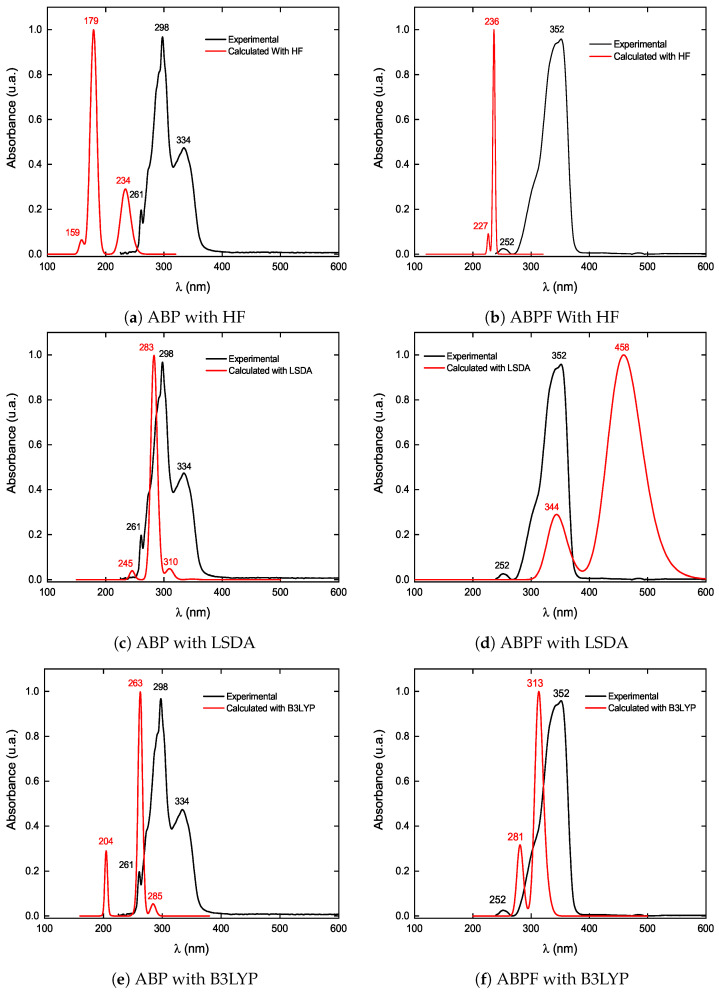
Comparison of the UV-Vis absorption spectra of ABP (**left-hand graph**) and ABPF (**right-hand graph**), experimental (black line) and theoretical (red line).

In Gaussian-16 [30], the exchange-correlation functionals LSDA and B3LYP were used, with the basis set 6-311 + G(2d,p), an SCF tolerance of $1.00\times 10^{-6}$ eV/atom and 204 cycles, to calculate the quantum shielding of ^1^H, ^13^C, ^17^O and ^11^B, in ABP and ABPF. To obtain the coupling constants of the hydrogen atoms H7, H8, H9, and H10 of the phenyl ring of ABP, the Hartree–Fock (HF) and DFT levels of theory were used with the LSDA and B3LYP functionals. The results of applying this functional can be seen in the Figure 4, Figure 5, Figure 6, Figure 7 and Figure 8, and Table 5, Table 6 and Table 8 of the Section 3.1.

#### 2.2.4. UV-Vis Absorption Spectra for ABP and ABPF

The UV-vis absorption spectra were calculated with the software Gaussian-16 [30] from the levels theory Hartree–Fock (HF) [43], configuration interaction (CI) [44], and time-dependent DFT (TD-DFT) [45] with 10 excited states in the singlet configuration, using the 6-311 + G(2d,p) basis set and the exchange-correlation functionals APFD, B3LYP, B3PW91, BVP86, CAM-B3LYP, HCTH, HSEH1PBE, LSDA, MPW1PW91, PBEPBE, and TPSSTPSS. In previous works, it is indicated that hybrid functionals such as B3LYP work very well on organic molecules [46], take into account the effect of moderate electron correlation between their atoms, spectral properties and minimization of systematic errors when compared to various databases [47]. The results of applying these functionals can be seen in the Table 9, Table 10 and Table 11 and some functionals in the Figure 8 of the Section 3.2.

#### 2.2.5. Infrared of ABP and ABPF

The frequency calculations for ABP and ABPF in their fundamental states were performed by means of Gaussian-16 [30], using the B3LYP/6-311 + G(2d,p) level of theory [36,37,38]. The energy at the zero point was calculated by scaling the harmonic frequencies by a factor of 0.965 [48,49,50]. The results of applying this functional can be seen in the Figure 11 and Figure 12 of the Section 3.3.

## 3. Results and Discussion

### 3.1. NMR for ABP and ABPF

The structural parameters of the ABP and ABPF were obtained by their geometric optimization using the exchange-correlation functionals PBE and B3LYP, as shown in Table 1 and Table 2.

**Table 1:** C–H bond lengths (Å) reported by X-ray diffraction analysis [51], and optimized by Tanış et al. [52] for the structure of the ABP compound. In this work, the optimization was performed for the ABP and ABPF compounds, using the exchange-correlation functionals PBE and B3LYP.

Bond	ABP		ABP (This Work)		ABPF (This Work)	
	**X-rays** [51]	**Tanış et al. [52]**	**PBE**	**B3LYP**	**PBE**	**B3LYP**
C1–H1	0.96	1.07	1.10	1.09	1.10	1.09
C2–H2	0.96	1.18	1.10	1.09	1.10	1.09
C3–H3	0.96	1.07	1.10	1.09	1.09	1.09
C4–H4	0.96	1.07	1.10	1.09	1.10	1.09
C7–H7	0.93	1.07	1.09	1.08	1.09	1.08
C8–H8	0.93	1.07	1.09	1.08	1.09	1.08
C9–H9	0.93	1.07	1.09	1.08	1.09	1.08
C10–H10	0.93	1.07	1.09	1.08	1.09	1.08
C13–H13	0.93	1.03	1.12	1.11	1.12	1.11

**Table 2:** C–C bond lengths (Å) and dihedra angle C9–C12–B1–O1 (°) reported by X-ray diffraction analysis [51], and optimized by Tanış et al. [52] for the ABP compound. In this work the optimization was performed for the ABP and ABPF compounds, using the exchange-correlation functionals PBE and B3LYP.

Bond	ABP		ABP (This Work)		ABPF (This Work)	
	**X-rays** **[51]**	**Tanış et al. [52]**	**PBE**	**B3LYP**	**PBE**	**B3LYP**
C1–C5	1.50	1.54	1.53	1.52	1.53	1.53
C2–C5	1.50	1.54	1.53	1.53	1.56	1.54
C3–C6	1.51	1.54	1.53	1.52	1.53	1.53
C4–C6	1.49	1.54	1.53	1.53	1.56	1.54
C5–C6	1.57	1.53	1.61	1.58	1.60	1.58
C7–C9	1.38	1.40	1.40	1.38	1.39	1.38
C7–C11	1.38	1.40	1.41	1.40	1.41	1.38
C8–C10	1.38	1.40	1.39	1.40	1.39	1.39
C8–C11	1.38	1.40	1.41	1.40	1.41	1.40
C9–C12	1.39	1.40	1.41	1.41	1.41	1.41
C10–C12	1.39	1.40	1.41	1.40	1.41	1.40
C11–C13	1.48	1.54	1.48	1.48	1.47	1.46
C13–O3	1.20	1.50	1.22	1.21	1.22	1.22
B1–O1	1.35	1.54	1.37	1.37	1.47	1.47
B1–O2	1.35	1.54	1.37	1.37	1.50	1.47
B1–C12	1.55	1.65	1.56	1.56	1.62	1.63
**Angle**						
C9–C12–B1–O1	8.33	-	9.86	1.96	27.78	24.83

The carbon–hydrogen bond lengths in the ABP compound exhibit a slight variation from the optimized structure: from 0.13 to 0.16 Å for the PBE and B3LYP functionals, compared to the experimental values (see Table 1 and Figure 1). This difference could be due to the greater freedom of movement of the hydrogen atoms in the ABP compound, which can not be interpreted in the frame of DFT. However, the carbon–carbon bond lengths exhibit even smaller variations: from 0.00 Å to 0.04 Å for PBE and B3LYP functionals, concerning the experimental values (see Table 2 and Figure 1), which could be due to the carbon atoms having stronger bonds. On the other hand, we must discuss the change that occurs when the F ion binds to the ABP compound to become the ABPF compound. The variation of the carbon–carbon and carbon–hydrogen bonds between ABPF and ABP do not suffer many changes. However, if we can appreciate a variation in the boron–oxygen and boron–carbon bonds given in Table 2, B1–O1 (0.10 Å), B1–O2 (0.10 Å) and B1–C12 (0.08 Å), where there is an increase in bond length due to the transformation of the ABP compound by the presence of the fluorine atom (ABPF anion). The atomic coordinates of the optimized ABP and ABPF structures in this work can be found in the supporting information.

Some values of the bond lengths reported by Tanış and co-workers [52] present a variation with respect to the values reported by X-ray diffraction [51] and those calculated in our work. For example, the bonds obtained by Tanış et al. [52] C13–O3, B1–O1, B1–O2, and B1–C12 are elongated compared to the experimental bond lengths and those optimized by the PBE and B3lYP functionals (see Table 2).

Table 3 and Table 4 show the experimental and optimized bond lengths for the compounds Si(CH_3_)_4_ and BF_3_·OEt_2_, which had variations from 0.04 to 0.76 Å. The contrast of the optimized bond lengths with respect to those reported by single crystal X-ray diffraction [51,53,54] are not significant enough to warrant further investigation into the structure of the reference compounds (Table 3 and Table 4); therefore, geometric optimization methods applied to traverses of the Gaussian-16 and CASTEP packages are valid for the reference isotropic shielding calculations.

**Table 3:** C–H and C–Si bond lengths (Å) reported by X-ray diffraction analysis [53] and optimized, using the exchange-correlation functionals PBE and B3LYP for the reference compound Si(CH_3_)4.

Bond	X-rays [53]	PBE	B3LYP
C–H	0.91	1.11	1.09
C–Si	1.85	1.89	1.89

**Table 4:** C–H, C–C, O–C, O–B and F–B bond lengths (Å) reported by X-ray diffraction analysis [54] and optimized, using the exchange-correlation functionals PBE and B3LYP for the reference compound BF_3_·OEt_2_.

Bond	X-rays [54]	PBE	B3LYP
C–H	0.98	1.10	1.09
C–C	1.34	1.52	1.51
O–C	1.41	1.50	1.48
O–B	1.56	1.67	1.61
F–B	1.33	1.41	1.37

The calculated electronic energy of the ABP conformer of the compound 4-(4,4,5,5-Tetramethyl-1,3,2-dioxoborolan-2-yl)benzaldehyde was −756.50 Ha. The difference in some structural parameters mentioned above (Table 2) exposes the energy differences reported by Tanış et al. [52] and the one calculated in this work. The ABP conformer, is 0.03 Ha more stable than the A2 taken by Tanış et al. [52], to determine the electronic and spectroscopic properties of the compound 4-(4,4,5,5-Tetramethyl-1,3,2-dioxoborolan-2-yl)benzaldehyde.

From the optimized structures of the reference compounds Si(CH_3_)_4_ and BF_3_·OEt_2_, we observe that the NMR Chemical shift ^13^C, ^1^H and ^11^B obtained in this work, are consistent with those reported in the literature by other authors (see Table 5) [55,56,57,58].

Before analyzing the results of the NMR theoretical calculations for the compound ABP, let us comment that all the labels for hydrogen and carbon atoms are indicated in Figure 1.

**Table 5:** Chemical shift (ppm) ^1^H, ^13^C for Si(CH_3_)_4_ and ^11^B for BF_3_·OEt_2_.

Reference	Method	^1^H	^13^C	^11^B
This work	CASTEP GIPAW PBE (DFT-D)	30.08	180.54	91.49
	Gaussian-09 B3LYP	31.93	183.77	99.77
[55]	CASTEP GIPAW PBE (DFT-D)	30.58	179.40	88.08
	Gaussian-09 B3LYP	31.93	183.77	99.77
[56]	Gaussian-94 B3LYP/6-311	31.28	179.15	-
[57]	Gaussian-94 B3LYP/6-311	31.88	182.47	-
[58]	Q-Express GIPAW	30.80	179.33	-

Figure 4 illustrates the magnetic resonance spectrum of the hydrogen atoms in the ABP compound, showing the experimental peaks (black line) and shielding calculated with the functionals, B3LYP (red line), LSDA (blue line), PBE with DFT + D (green line). The resonance peaks of the H1–H4 atoms (0.00–1.50 ppm) of the methyl groups are at high field because they are shielded. However, the resonance peaks of the H7–H10 atoms (6.80–8.40 ppm) are at low field, because they are poorly shielded, due to the delocalization of the *π* electrons on the phenyl ring. The resonance peak of the H13 atom (10.12–10.50 ppm) is at low field, due to the unshielding caused by the inductive effect of the oxygen atom of the carbonyl group on the carbon atom attached to the H13 hydrogen atom.

Figure 5 illustrates the resonance spectra of the carbon atoms of the ABP compound with experimental data and theoretical results, with the same color distribution for the methods given in Figure 4. It can be seen from the two figures that the theoretical data reported with B3LYP in Gaussian-16 (red line) are very close to the experimental data. The resonance peaks of the C1–C4 atoms (24.92–27.97 ppm) of the methyl groups are at high field. However, the resonance peaks of the C5–C6 atoms (92.00–92.58 ppm) are at mid-field, due to the inductive influence of the oxygen atoms O1 and O2. The resonance peak of the C11 atom (143.99 ppm) is at low field, due to the inductive effect of the oxygen atom of the carbonyl group and C12 (143.92 ppm) due to the inductive effect of the boron atom. The resonance peak of the C13 atom (201.88 ppm) attached to the oxygen atom O3 is at low field, i.e., more unshielded, due to the oxygen atom attracting the higher electron density in the C=O double bond.

Table 6 shows in detail the chemical shifts of the hydrogen atoms in the compounds ABP and ABPF, plus a comparison with data reported by Tanış et al. [52], which are contrasted from the experimental data and those calculated by the PBE, LSDA, and B3LYP functionals, in compound ABP. From Table 6 and Figure 6, the difference of the theoretical shifts between compound ABPF and ABPF can be observed, where the changes are not so significant.

**Table 6:** Experimental (Exp.) NMR signals ^1^H (ppm) of ABP compounds and calculated shielding using the exchange-correlation functionals PBE, LSDA, and B3LYP, for the ABP and ABPF compounds.

Atom	ABP		ABP (This Work)			ABPF (This Work)		
	Exp.	Tanış et al. [52]	PBE	LSDA	B3LYP	PBE	LSDA	B3LYP
H1	1.34	0.72	1.26	1.44	1.37	1.20	1.17	0.97
H2	1.34	0.80	1.30	1.35	1.29	1.06	1.40	1.18
H3	1.34	0.70	1.26	1.44	1.38	1.20	1.07	1.04
H4	1.34	0.80	1.30	1.36	1.29	0.80	0.98	0.67
H7	7.84	7.36	7.96	8.36	8.38	7.88	8.01	8.10
H8	7.84	7.91	7.71	8.04	8.07	7.59	7.65	7.72
H9	7.94	7.50	7.84	8.40	8.33	7.73	8.58	8.09
H10	7.94	7.64	7.99	8.50	8.41	7.75	8.19	8.07
H13	10.03	14.69	10.75	10.68	10.48	10.22	10.44	10.29

The difference between the PBE and B3LYP functionals for chemical shielding is due to the different exchange-correlation functionals used (Table 6). PBE is a semi-local functional, while B3LYP is a hybrid functional. Semi-local functionals do not explicitly explain electron correlation, while hybrid functionals do. This difference in the treatment of the electron correlation is probably responsible for the difference in the calculated chemical shielding values. In general, we can say that the functional that best fits the experimental calculations is the B3LYP, compared to the PBE and LSDA functionals.

Table 7 shows the neighborhood coupling constants, ^3^*J*_*H*−*H*_, where the typical values of the nuclei of the hydrogen atoms in position *ortho* (1,2) in a phenyl ring are between 6–10 Hz [7]. Both the experimental data and the results of the B3LYP functional for the spin–spin coupling constant are within the expected values. The coupling constant can be used to determine the relative orientation of the two protons. For example, if the coupling constant is between 6–10 Hz, then the two protons would be in the position *ortho* (H7 and H9 or H8 and H10, see Figure 1) or if the coupling constant is between 1–3 Hz, then the two protons would be in the position *meta* (1,3) in the phenyl ring [7]. The B3LYP functional gives a good match for the spin-spin coupling constant of the hydrogen atoms of the phenyl ring of the ABP compound, while the calculation for the coupling constant with LSDA is far from the expected values and with the HF level of theory no coupling of the phenyl ring nuclear spins is predicted. This marked difference is due to the fact that HF has no electronic correlation and the DFT methods do.

**Table 7:** Experimental and calculated spin-spin coupling constants, ^3^*J*_*H*−*H*_ (Hz), for the nuclei of the hydrogen atoms H7, H8, H9 and H10 of the compound ABP.

Coupling	Experimental	Calculated		
		B3LYP	LSDA	HF
^3^ *J*_*H*7−*H*9_	8.22	7.06	5.50	−0.49
^3^ *J*_*H*8−*H*10_	7.06	6.78	5.29	−1.10

Now, let us analyze the chemical shifts as a function of the carbon atoms in the NMR spectrum of the compound ABP [59]. The carbon atoms are labeled in Figure 1.

Table 8 shows in detail the chemical shifts of the carbon atoms of ABP compound, including a comparison with data reported by Tanış et al. [52], which are contrasted from the experimental data and those calculated by the PBE, LSDA, and B3LYP functionals, in the ABP compound. Figure 7 also shows the difference in the theoretical shifts between the ABPF and ABP compounds, where the shifts are very significant for the C12, O1, and O2 atoms, as well as for B1 when the fluoride ion is covalently bonded to the ABP compound. The large low-field chemical shift of C12 indicates a large unshielding of this atom, which cannot be well taken into account for any exchange-correlation functional. Similarly, the high-field chemical shifts of the O1, O2, and B1 signals show a substantial increase in the shielding of these atoms. This is due to the inductive effect of the fluorine atom in the B–F bond in the ABPF compound (Figure 2). On the other hand, some chemical shifts of hydrogen (H13) and carbon (C11, C12, C13) reported by Tanış et al. [52] in the ABP compound show significant variation concerning experimental data and those calculated by the PBE, LSDA, and B3LYP functionals in this work.

It should be pointed out that Tanış et al. [52] have studied and compared the optimized structure of the A2 confomer of the compound 4-(4,4,5,5-Tetramethyl-1,3,2-Dioxoborolan-2-yl)benzaldehyde, with the previously reported optimized structure of 4-formylphenylbenzaldehyde, although the crystallographic data of the compound had already been published by Urdaneta et al. [51].

**Table 8:** Experimental (Exp.) ^13^C and ^11^B NMR signals (ppm) [59] of the ABP compound and calculated for the shielding of the nuclei of the isotopes of ^13^C, ^17^O and ^11^B, through the exchange-correlation functionals PBE, LSDA, and B3LYP for the ABP compound and ABPF anion.

Atom	ABP		ABP (This Work)			ABPF (This Work)		
	Exp. [59]	Tanış et al. [52]	PBE	LSDA	B3LYP	PBE	LSDA	B3LYP
C1	25.10	20.46	31.73	24.58	24.92	28.46	27.50	26.84
C2	25.10	24.11	30.94	27.97	27.83	31.61	28.59	28.39
C3	25.10	20.43	31.66	24.62	25.03	31.67	27.70	26.95
C4	25.10	23.98	31.00	28.03	27.83	28.98	28.33	27.64
C5	84.50	85.71	99.16	90.40	92.58	93.26	82.40	84.58
C6	84.50	85.80	99.42	90.45	92.58	94.51	84.04	85.70
C7	128.80	126.11	136.26	131.87	130.85	136.10	128.72	128.94
C8	128.80	132.83	145.68	142.92	140.46	146.51	142.01	140.05
C9	135.00	133.49	147.12	142.55	141.23	144.38	140.59	136.94
C10	135.00	136.06	147.30	143.01	141.58	145.09	140.66	137.51
C11	138.50	163.29	149.64	143.17	144.01	145.47	138.95	138.12
C12	138.00	147.57	148.77	144.87	144.01	177.54	181.73	179.74
C13	192.90	349.60	207.63	207.12	201.88	207.83	204.58	201.15
O1	-	-	194.00	201.80	183.65	116.48	124.66	114.41
O2	-	-	193.19	201.35	183.31	123.51	144.57	115.22
B1	31.10	-	29.98	27.46	27.57	3.65	3.08	4.06

Energy variations in the calculations may be indicative of structural rearrangements or changes in the geometric and electronic configurations of the compounds studied. The relative energy values calculated for the different conformers provide information on their stability and their most likely configuration. Higher energy conformers may correspond to less stable structures, while lower energy conformers are more stable. Structural parameters, such as bond lengths and angles, can be affected by energy variations. The energy differences between the conformers can be attributed to variations in electronic energy, which may be due to changes in molecular orbital energies and electronic interactions. These energy variations and their correlation with structural rearrangements or electronic configurations are crucial for understanding the stability and properties of the compounds studied.

### 3.2. UV-Vis of ABP and ABPF

In this section, we perform a detailed comparison between the experimental absorption spectra and the calculated spectra by computational methods HF, LSDA, and B3LYP for the compounds, ABP and ABPF. The experimental measurements (black lines) are contrasted with the results obtained through computational methods (red lines) from Figure 8a–f. For the compound ABP, the comparisons are presented in Figure 8a,c,e, while for ABPF, they are detailed in Figure 8b,d,f.

In the absorption spectrum of the ABP compound, the wavelengths in 334 nm correspond to the electronic transition *n* → π*, and the 298 nm and 261 nm to the transitions *π* → π*. For the absorption spectrum of the ABPF anion, the wavelengths 352 nm and 252 nm correspond to the transition *π* → π*. In the absorption spectrum of the ABPF anion, the electronic transition *n* → π* is absent (the wavelengths in 334 nm) and the transitions *π* → π* is shifted by 54 nm towards higher wavelength values (bathochromic shift), due to the coordination of the boron atom with the fluoride anion.

Table 9 contains the values of the experimental and calculated wavelengths with their respective absolute variations. They correspond to the UV-vis region of the electromagnetic spectrum, considering the levels theory HF, CI, and TD-DFT for the absorption spectrum of the ABP compound and the ABPF anion. It is observed that the wavelengths calculated with HF (see Figure 8) for the absorption bands were far from the experimental ones, however, the B3LYP functional with the DFT level of theory had the best approximation of the values of the wavelengths of the absorption bands in the UV-vis spectrum for the ABP compound and the ABPF anion.

Table 10 summarizes the experimental (Exp.) and calculated absorption band wavelengths, the electron promotion energy, the oscillator strength, and the levels of the molecular orbitals associated with the electron transitions. The calculated values were obtained using the HF and DFT levels of theory with the exchange-correlation functionals LSDA and B3LYP, for the compounds ABP and ABPF. All the calculation methods reproduce the same order of occurrence of the electronic transitions as the experimental spectra (i.e., *π* → π* < *n* → π* as a function of λ in Figure 8). The calculation of the λ_*max*_ absorption for the ABP compound and the ABPF anion had a better approximation with the B3LYP functional.

**Figure 9:**
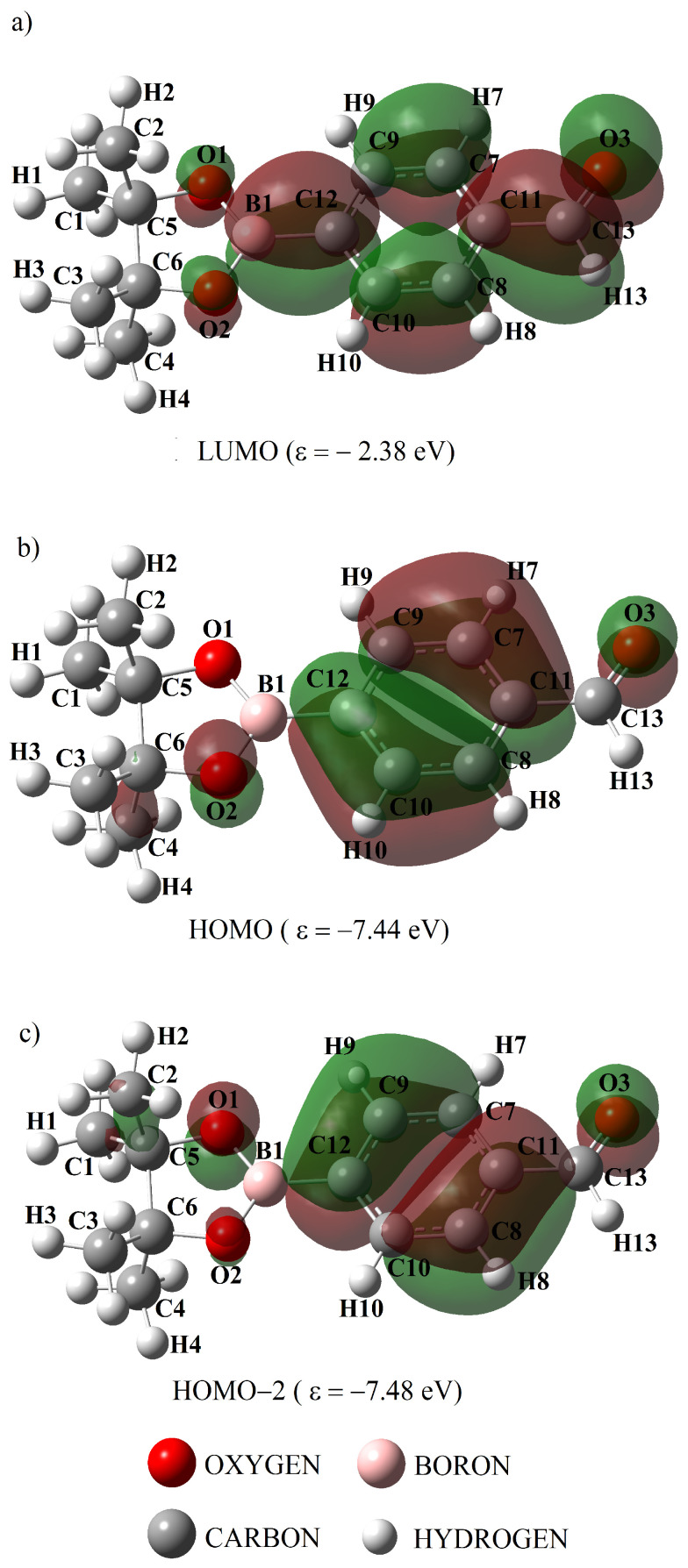
Molecular orbitals calculated with the B3LYP level of theory, related to the highest intensity electronic transitions in the UV-vis region of the ABP compound. Panel (**a**) represent LUMO, while panels (**b**,**c**) the corresponding HOMO for two different energies.

**Figure 10:**
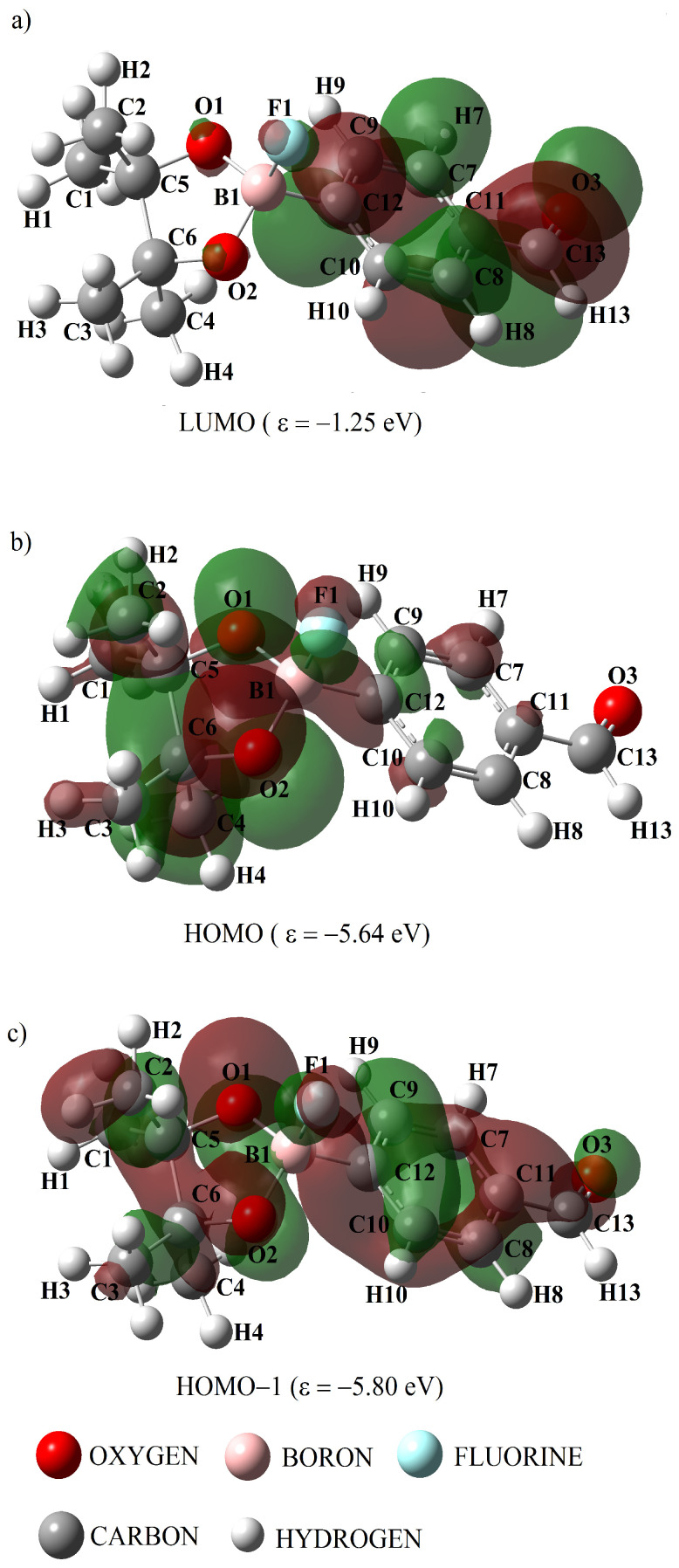
Molecular orbitals calculated with the B3LYP level of theory, related to the highest intensity electronic transitions in the UV-vis region of the ABPF anion. Panel (**a**) represent LUMO, while panels (**b**,**c**) the corresponding HOMO for two different energies.

Figure 9 and Figure 10 show the molecular orbitals calculated with the B3LYP theory level, which are related to the electronic transitions *π* → π* and *n* → π*, for the compounds ABP and ABPF. The red color indicates a positive phase of molecular orbitals, while their negative phase is indicated by the green color. At 204 and 263 nm (Table 10), the DFT method predicts that the highest photon absorption occurs, through the transitions *π* → π* in H → L + 1 and H − 2 → L, respectively. Meanwhile, at 285 nm the transitions *n* → π* in H → L for the ABP compound (see Table 10). It can be seen in Figure 9a–c that for the compound ABP there is a large participation of the carbon atoms of the phenyl ring in the orbitals *π*, π*, extending up to the boron atom (Figure 9a) and the oxygen atoms O1, O2 and O3 in the orbitals *n*, π*.

In the supporting information, Table S5 indicates the Natural Bond Orbital (NBO) analysis, detailing the electronic occupancy, orbital bond, molecular orbital coefficients, and hybrid orbitals of the atoms C11, C13, H13, and O3 of the formyl group of the ABP compound. For each NBO, the label “BD” represents a 2-center bond, “LP” is labeled for a lone pair of valence electrons from a 1-center, and “BD*” for a 2-center antibond. Labels without asterisks and with asterisks correspond to Lewis and non-Lewis NBOs, respectively, where the numbering 1 and 2 is placed if there is a single or double bond, respectively, between a pair of atoms. Table S5 shows the natural hybrid atomics hA that make up the NBO, with the percentage (100$c_{A}^{2}$) of the NBO in each hybrid (in parenthesis), the atom label, and a hybrid label showing the sp composition (percentage of s character, p character, and d character) of each h_A_. This analysis is carried out by examining all possible interactions between ‘filled’ Lewis-type NBOs (donors) and ‘empty’ non-Lewis NBOs (acceptors) and estimating their energetic importance using second-order perturbation theory. Since these interactions lead to the loss of occupancy from the localized NBOs of the idealized Lewis structure to the empty non-Lewis orbitals (and, therefore, deviations from the idealized Lewis structure description), they are known as “delocalization” corrections to the zero or natural Lewis structure order. For each donor NBO (i) and acceptor NBO (j), the stabilization energy E(2) associated with delocalization (“2e stabilization”) is estimated. It can be seen in Table S5 that although interactions between BD(1) C13–O3 bonds and BD*(1) (C11–C8 and C11–C13) bonds (1.22 and 1.09 kcal/mol), BD(2) C13–O3 bonds and BD*(2) (C11–C8) bonds (5.06 kcal/mol), which can be associated with σ → σ* and *π* → π* transitions, the strongest interactions are identified for the interaction between lone pair orbitals LP(2) located on oxygen atom O12 with adjacent BD*(1) (C11–C13 and C13–H13) bonds (17.96 and 23.15 kcal/mol), which can be associated with *n* → σ* transitions.

**Table 9:** Wavelength of UV-vis spectra calculated using different exchange-correlation functionals.

Method	ABP		ABPF	
	Theoretical	Difference with Experiment	Theoretical	Difference with Experiment
	λ ** (nm)**	(nm)	λ ** (nm)**	(nm)
B3LYP	204	57	281	29
	263	35	312	40
	285	49		
HF	159	102	227	25
	179	119	236	116
	234	100		
APFD	201	60	275	23
	258	40	305	47
	279	55		
B3PW91	203	58	279	23
	261	37	316	47
	283	51		
BVP86	242	19	335	83
	282	16	444	92
	307	27		
CAM-B3LYP	190	71	242	10
	250	48	261	91
	265	69		
HCTH	240	21	330	78
	281	17	441	89
	306	28		
HSEH1PBE	201	60	274	22
	258	40	308	44
	279	55		
LSDA	245	16	344	92
	283	15	458	76
	310	24		
MPW1PW91	200	61	273	21
	257	41	279	55
	277	57		
PBEPBE	243	18	338	86
	282	16	450	98
	307	27		
TPSSTPSS	232	29	319	67
	275	23	417	65
	301	33		
CI	159	102	223	29
	174	124	230	23
	228	106		

At 313 nm (see Table 11) the highest photon absorption occurs through the *π* → π* transitions in H − 1 → L, while the band corresponding to the *n* → π* transitions does not appear for the ABPF anion (see Table 10 and Figure 8). Figure 10 shows the change of the HOMO orbital due to the presence of the fluorine atom in the ABPF anion, where the carbon atoms of the phenyl ring and the carbonyl group do not participate. It can also be observed that the π* orbitals of the carbon atoms of the phenyl ring of the ABPF anion, do not extend to the boron atom in the LUMO orbital (see Figure 9a and Figure 10a) as in the ABP compound, i.e., the boron atom does not participate in the formation of the LUMO orbital of the ABPF ring. This marked difference between the molecular orbitals could be linked to the change in the optoelectronic response of the ABPF anion with respect to the ABP compound, where there is an increase in λ_*max*_ and a decrease in the electronic energy gap between its boundary orbitals, transition dipole moment and quantum yield (see Table 11).

Experimental and theoretical analysis of ABP and ABPF compounds reveals changes in their absorption and fluorescence spectra, indicating changes in electronic transitions due to the presence of the fluorine atom in the ABP compound. Theoretical calculations that use different exchange and correlation functionals, such as PBE and B3LYP, provide information on the electronic energy and structural rearrangements that occur when the boron atom bonds with the fluoride ion. The shift in the peaks of the ^13^C, ^11^B and ^17^O NMR spectra of the ABP compound (see Table 8 and Figure 7), due to the covalent bonding of the fluoride ion, indicates that there is great shielding at the B1 boron atom and the oxygen atoms (O1 and O2) that bind to it, while in contrast there is a large unshielding of the C12 carbon atom of the phenyl ring occurs, attached to the boron atom. This leads to the assumption that the electron density during the formation of the B–F bond increases in the B1, O1, and O2 atoms, and decreases in the C12 carbon atom.

**Table 10:** Experimental (Exp.) and calculated absorption wavelength λ (nm), electronic promotion energy ε (eV), or energy levels of the promotion states and oscillator strength f, using the HF and DFT levels of theory with the exchange-correlation functionals LSDA and B3LYP for the compounds ABP and ABPF.

	Trans.	λ	ε	f	Trans.	λ	ε	f
	ABP				ABPF			
Exp.		334	3.72			352	3.53	
		298	4.16			252	4.93	
		261	4.76					
B3LYP	H → L	285	4.35	0.0346	H − 1 → L	313	3.97	0.0949
	H − 2 → L	263	4.72	0.6411	H − 3 → L	281	4.42	0.0313
	H → L + 1	204	6.08	0.1864				
LSDA	H − 3 → L	310	4.00	0.0291	H − 1 → L	458	2.71	0.0556
	H − 2 → L	283	4.39	0.6072	H − 3 → L	344	3.61	0.0157
	H − 1 → L + 1	245	5.07	0.0234				
HF	H → L + 1	234	5.30	0.2489	H − 1 → L + 5	236	5.26	0.03499
	H − 1 → L + 1	179	6.93	1.1305	H − 1 → L + 5	227	5.44	0.0320
	H → L + 2	159	7.81	0.0677				

Trans.: Electronic transition between molecular orbitals. H: HOMO. L: LUMO.

**Table 11:** Experimental (E) and calculated (T) maximum absorption wavelength $\lambda_{max}$ (nm), energy gap between the boundary molecular orbitals Δ(H–L) (eV), transition dipole moment $\mu_{T}$ (Debye) and quantum yield $\Phi_{F(X)}$, using the DFT level of theory with the exchange-correlation functional B3LYP for the ABP compound and ABPF anion.

$\lambda_{\max}$ (E)	$\lambda_{\max}$ (T)	Δ(H–L)	$\mu_{T}$	$\Phi_{F(X)}$	$\lambda_{\max}$ (E)	$\lambda_{\max}$ (T)	Δ(H–L)	$\mu_{T}$	$\Phi_{F(X)}$
**ABP**					**ABPF**				
298	263	5.06	2.35	0.27	352	313	4.38	0.99	0.01

H: HOMO. L: LUMO.

During the formation of the B–F bond, the rearrangement of the electronic density occurs in the B1 boron atom and its neighboring atoms (C12, O1, and O2), due to the inductive effect of the fluorine atom, producing the shift of the spectrum absorption in the UV-vis at longer wavelengths (batochromic), a decrease in the energy gap of the frontier orbitals Δ(H–L) and decrease in the transition dipole moment (*μ_T_*) (Table 11). The fluorescence spectra also show changes in the emission peaks, suggesting changes in the excited state properties of the compound ABP when is covalently coordinated with fluorine ions. As in the absorption spectrum, the decrease in the energies of the frontier molecular orbitals Δ(H–L), the decrease in the transition dipole moment (*μ_T_*), the decrease in the quantum yield (Φ_*F*(*X*)_) (Table 11) and the rearrangement of the electron density in the boron atom B1 like that of its neighboring atoms (O1, O2, and C12) during the formation of the B–F bond, cause the decrease in intensity in the fluorescence spectrum of the ABPF anion.

The rearrangement of the molecular orbitals of ABP and ABPF can be seen in Figure 9 and Figure 10, where we observe that the HOMO orbital located in the phenyl ring of ABP moves towards the heterocyclic ring where the B1 boron atom (BO_2_) is located when it is bound to the fluoride ion to form ABPF (see Figure 9b and Figure 10b).

The combination of experimental and theoretical analyses allows a complete understanding of the spectral changes and their correlation with the B–F bond. Changes in the electronic transitions, excited state properties, and electronic configurations of ABP and ABPF contribute to the observed changes. Theoretical calculations provide information on electronic energy and structural rearrangements, while experimental analysis confirms spectral changes.

**Figure 11:**
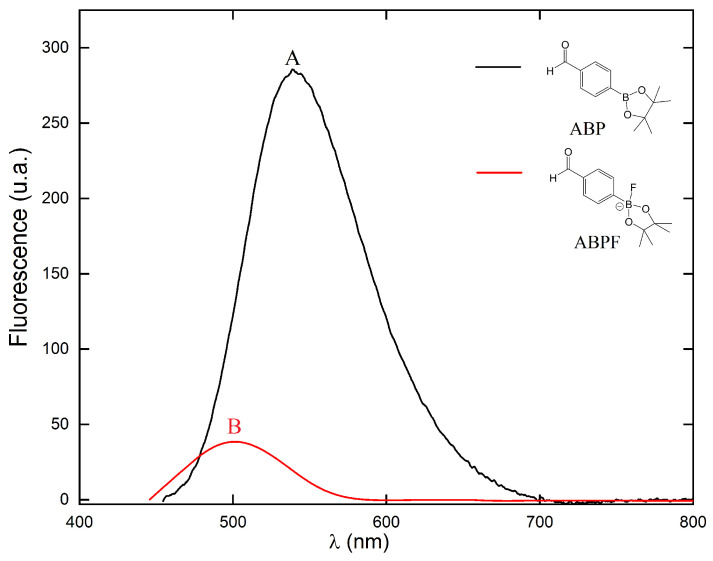
Experimental fluorescence spectra of the compounds ABP (black line) and ABPF (red line).

The decrease in the quantum yield Φ_*F*(*X*)_ in the ABP compound (see Table 11) is in agreement with previous work published by our research group, where the emission of the absorbed photons depends on the electronic nature of the groups attached to the molecule. The quantum yield increases for organic compounds containing electron-donating groups bonded in position on the phenyl ring, whereas, it decreases when the molecule has electron-attracting groups bonded in the same position *para* on the phenyl ring [60,61]. The fluorine atom has a double effect in the ABPF anion studied in this work. First, it increases the electronic density of the boron atom and its neighboring oxygen atoms (O1 and O2). Second, its inductive effect attracts part of the el ectron density of the *π* orbitals of the phenyl ring, with the C12 carbon atom of the ring being the most unshielded (see Figure 7). On the other hand, the formation of a Boron-Fluor covalent bond induces the energy absorbed by the electrons (radiative transition) of the ABPF in the UV-vis region to be emitted through non-radiative transitions (see Figure 8 and Figure 11).

It can be seen in Figure 11, that the fluorescence spectrum of the ABP compound decreases dramatically in intensity. Moreover, the photon emission peak at λ_*max*_ presents a 38 nm shift towards lower wavelength values (hypsochromic shift, A → B). This can be explained by the coordination of the fluoride anion with the boron atom of the ABP compound. These optoelectronic response changes in the UV-vis region of the ABP molecule are in agreement with those found in the literature for organic compounds containing the boronic acid functional group (-B(OH)_2_) and boronate ester (-B(OR)_2_) [62]. The experimental graph helps us understand the behavior of the ABP compound in the presence of the Fluoride ion.

### 3.3. Infrared of ABP and ABPF

**Figure 12:**
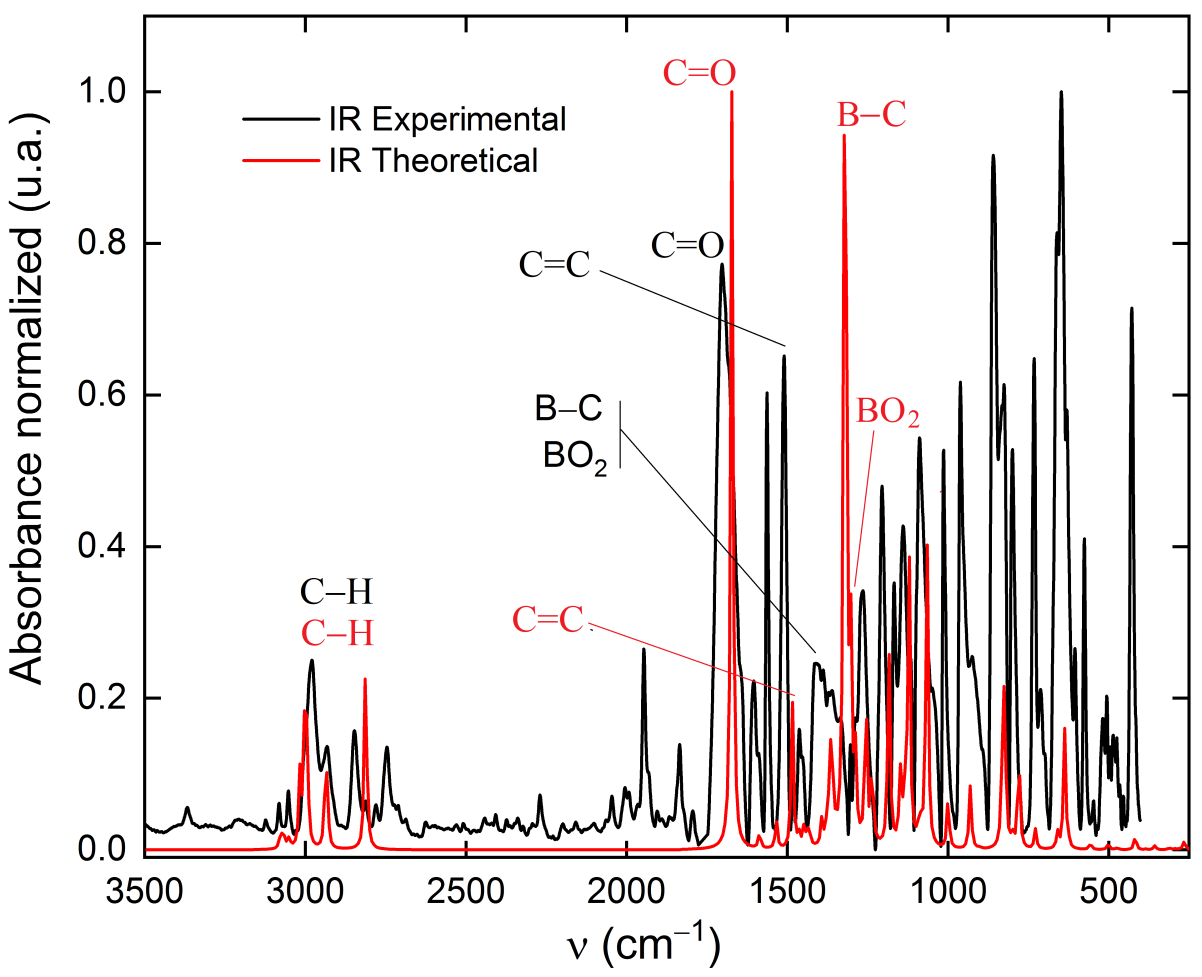
Experimental (black line) vs. theoretical (red line) infrared spectra for the compound ABP using the exchange-correlation functional B3LYP. The bands correspond to the characteristic stretching of some bonds of the ABP compound are: 2600 to 3100 cm^−1^ (C–H); 1650 to 1700 cm^−1^ (C=O); 1450 to 1550 cm^−1^ (C=C); 1300 to 1350 cm^−1^ (B–C and BO_2_).

Figure 12 shows the stretching absorption bands of some bonds of the ABP compound in the infrared spectrum. It is found that the theoretical spectrum obtained using the DFT level theory with the B3LYP functional (red line), has a good approximation for the most intense bond vibrational bands of the experimental spectrum (black line). The stretching absorption bands from 2938 to 2980 cm^−1^ are due to symmetric (*ν*_s_CH_3_) and asymmetric (*ν*_as_CH_3_) stretching of the C–H bonds of the methyl groups attached to the C5–C6 carbon atoms (see Figure 1) [63]. The stretching of the C–H bond of the formyl group is assigned to the band from 2810 to 2850 cm^−1^, which has a frequency similar to that reported for Dwivedi and Rai [64]. The absorbance peaks between 1675 to 1702 cm^−1^ indicate the stretching of the C=O bond vibration, evidencing the presence of the formyl group. The vibrations involving carbon–carbon stretching within the phenyl ring are absorbed in the regions from 1450 to 1550 cm^−1^. The strongest bands in the theoretical and experimental IR spectra, located in the peaks 1300 and 1350 cm^−1^, are assigned to the asymmetric stretching (*ν*_as_) of the B–C bond and to the bending asymmetric vibration scissoring of BO_2_ [65].

**Figure 13:**
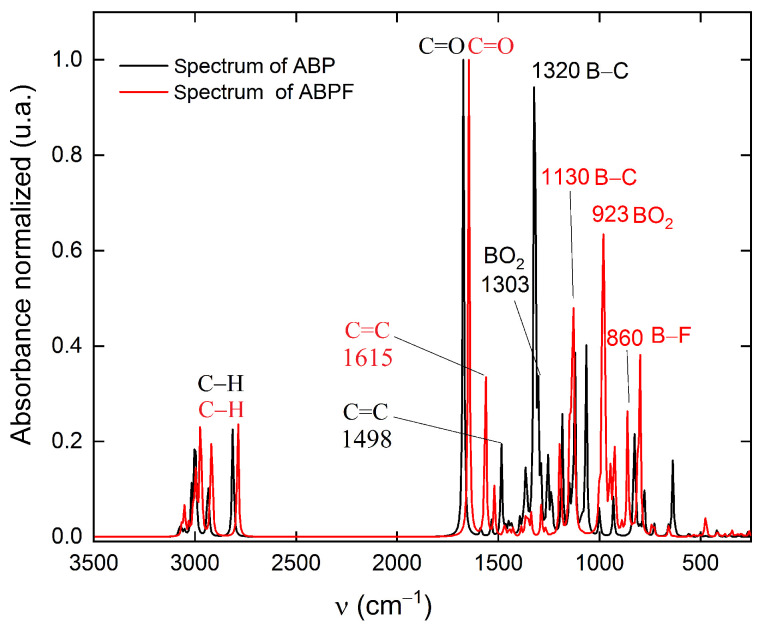
Infrared spectra calculated using the B3LYP functional for ABP (black line) and ABPF (red line).

Finally, it can be seen in Figure 13 that the calculated spectra of ABP and ABPF through the density functional theory using the B3LYP functional. When comparing the spectra of ABP (black line) and ABPF (red line) it can be observed that the stretches C–H and C=O do not present significant variations; however, the stretches C=C, B–C and the bending BO_2_ present shifts of the absorption bands. Evidently, the fluoride ion influences the energy of the absorption bands due to the coordination of the fluoride ion with the boron atom of the ABP compound. This is in agreement with the other optical responses studied.

## 4. Conclusions

The results of this study demonstrate that DFT is a powerful tool that can be used to study the structure and electronic properties of the boronate esters of the ABP compound and ABPF anion. The DFT level of theory using the B3LYP functional shows the best agreement with the experimental data. The B3LYP functional in the DFT frame is a powerful tool to predict the structure and NMR spectra of ^1^H, ^13^C, ^11^B, UV-vis and IR spectra of the compounds studied, which can be used to understand their bonds and interactions. In NMR spectroscopy, the coordination of fluorine with the boron atom modifies the shielding and unshielding of certain atoms, reflecting a redistribution of electronic density. The UV-vis absorption spectra exhibit a bathochromic shift in the *π* → π* transitions for the ABPF anion, underlining the impact of fluorine on the electronic structure of the compound and its light absorption characteristics. Moreover, IR spectroscopy highlights that, while the stretching vibrations of C–H and C=O remain largely unchanged after fluorination, the vibrations associated with C=C, B–C bonds, and the bending of BO_2_ undergo significant shifts in their infrared bands due to the coordination of fluorine. The theoretical approach using the B3LYP functional proves effective in predicting these changes and can be implemented for the design and structure study of new boronate compounds with desired properties. The boron atom of the ABP compound is a Lewis acid and allows the formation of stable anions by coordinating with fluoride ions, producing changes in their optical and spectral response. This feature gives the ABP compound the potential to be used as a marker compound of fluoride ions.

## Data Availability

The data presented in this study are available on request from the corresponding author.

## Supplementary Materials

The following supporting information can be downloaded at: https://www.mdpi.com/article/10.3390/ijms25095000/s1.
