# Supplementary material for: Optoelectronic Response to the Fluor Ion Bond on 4-(4,4,5,5-Tetramethyl-1,3,2-dioxoborolan-2-yl)benzaldehyde
Source: Int J Mol Sci. 2024 May 3;25(9):5000. doi: 10.3390/ijms25095000 (PMC11084352; doi:10.3390/ijms25095000)
Supplement: Supplementary file 1 [file ijms-25-05000-s001.zip › ijms-2902132-supplementary.pdf]

Supporting Information

Optoelectronic Response to the Fluor Ion Bond on 4-(4,4,5,5-Tetramethyl-1,3,2-dioxoborolan-2-yl)benzaldehyde

Ulises J. Guevara [1] , Jesús Núñez [2] , Laura M. Pérez [3,*] , Anton Tiutiunnyk [4] , Neudo Urdaneta [5] , Eduardo Cisternas [6] and David Laroze [1]

1 Instituto de Alta Investigación, Universidad de Tarapacá, Arica 1000000, Chile; uguevara@academicos.uta.cl (U.J.G.)

2 Departamento de Biología, Universidad Politécnica Territorial del Oeste de Sucre "Clodosbaldo Russian", Cumaná 6101, Venezuela

3 Departamento de Ingeniería Industrial y de Sistemas, Universidad de Tarapacá, Arica 1000000, Chile

4 Departamento de Física, FACI, Universidad de Tarapacá, Arica 1000000, Chile

5 Departamento de Química, Universidad Simón Bolívar (USB), Caracas 1020-A, Venezuela

6 Departamento de Ciencias Físicas, Universidad de La Frontera, Casilla 54-D, Temuco 4811230, Chile; eduardo.cisternas@ufrontera.cl

* Correspondence: lperez@academicos.uta.cl

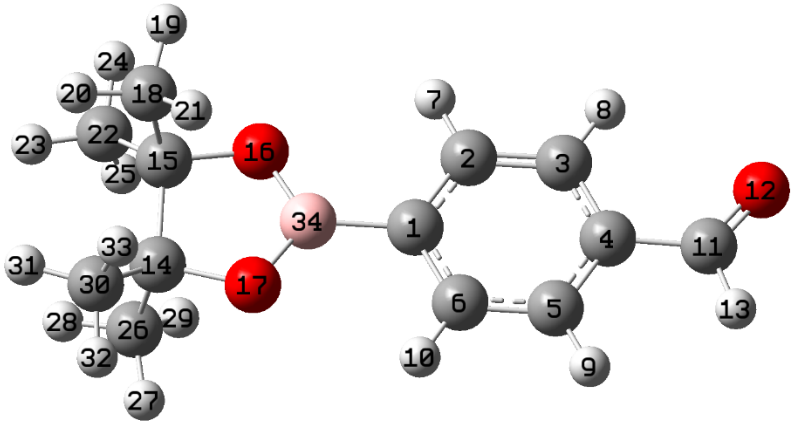

Figure S1. ABP compound structure optimized with B3LYP/6-311+G(2d,p) through Gaussian 16 software.

Table S1. Atomic coordinates of the **ABP** compound structure optimized with B3LYP/6-311+G(2d,p) through Gaussian 16 software.

N°	Atom	x	y	z
1	C	0.95534	0.08597	-0.00889
2	C	1.71725	-1.08696	0.13230
3	C	3.10121	-1.04616	0.12781
4	C	3.76081	0.17951	-0.02017
5	C	3.01719	1.35393	-0.16199
6	C	1.62951	1.30537	-0.15558
7	H	1.20982	-2.03758	0.24584
8	H	3.68767	-1.94999	0.23675
9	H	3.53150	2.30223	-0.27630
10	H	1.05952	2.22017	-0.26475
11	C	5.23386	0.25900	-0.02958
12	O	5.98473	-0.68921	0.08323
13	H	5.64175	1.28148	-0.15108
14	C	-2.77759	0.74063	0.09104
15	C	-2.72347	-0.82673	-0.08304
16	O	-1.31457	-1.12079	0.19140
17	O	-1.39431	1.13208	-0.19260
18	C	-2.99387	-1.29324	-1.51428
19	H	-2.75475	-2.35499	-1.59079
20	H	-4.04270	-1.15957	-1.78399
21	H	-2.37846	-0.75381	-2.23576
22	C	-3.57989	-1.61461	0.89836
23	H	-4.63611	-1.36756	0.77072
24	H	-3.45939	-2.68310	0.71189
25	H	-3.29863	-1.41612	1.93126
26	C	-3.07047	1.1877990	1.52401
27	H	-2.90447	2.26356	1.59900
28	H	-4.10592	0.98247	1.80040
29	H	-2.41485	0.69229	2.24155
30	C	-3.69306	1.46695	-0.88457
31	H	-4.72878	1.14743	-0.75005
32	H	-3.64579	2.54126	-0.69855
33	H	-3.40553	1.28819	-1.91934
34	B	-0.60449	0.03163	-0.00308

Table S2. Atomic coordinates of the **ABP** compound structure optimized with PBE through CASTEP software.

N°	Atom	x	y	Z
1	C	5.70252	5.82780	8.59590
2	C	5.74848	4.64100	7.83306
3	C	5.73780	4.67784	6.44436
4	C	5.67252	5.91228	5.77890
5	C	5.63436	7.10268	6.52212
6	C	5.65272	7.06008	7.91550
7	H	5.78796	3.67920	8.34606
8	H	5.77188	3.76908	5.84046
9	H	5.58468	8.05812	5.99148
10	H	5.61828	7.87560	8.48898
11	C	5.62800	5.97336	4.30092
12	O	5.63844	5.00880	3.54852
13	H	5.57616	7.01328	3.88836
14	C	5.68488	6.46524	12.35196
15	C	5.62008	4.85928	12.27708
16	O	5.52684	4.58808	10.84518
17	O	5.82384	6.86868	10.95480
18	C	4.38180	4.24236	12.92670
19	H	4.40652	3.15060	12.80628
20	H	4.33920	4.46352	14.00130
21	H	3.46176	4.60920	12.45492
22	C	6.88560	4.15608	12.77496
23	H	7.06608	4.35924	13.83858
24	H	6.77532	3.06996	12.65184
25	H	7.76952	4.46808	12.20364
26	C	6.90264	7.01544	13.09338
27	H	6.88380	8.11380	13.07322
28	H	6.91308	6.69612	14.14836
29	H	7.83612	6.68892	12.61836
30	C	4.40340	7.12332	12.87018
31	H	4.18968	6.82488	13.90482
32	H	4.51596	8.21616	12.84894
33	H	3.53868	6.86520	12.24468
34	B	5.68704	5.76312	10.15542

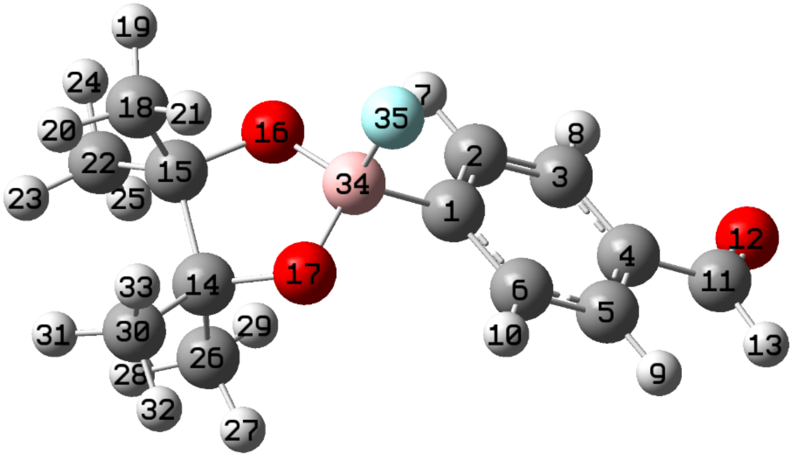

Figure S2. ABPF compound structure optimized with B3LYP/6-311+G(2d,p) through Gaussian 16 software.

Table S3. Atomic coordinates of the **ABPF** anion structure optimized with UB3LYP/6-311+G(2d,p) through Gaussian 16 software.

N°	Atom	x	y	z
1	C	-0.89467	0.03138	0.49867
2	C	-1.61722	-1.11442	0.11447
3	C	-2.97071	-1.06958	-0.17521
4	C	-3.66565	0.14589	-0.09220
5	C	-2.96626	1.30021	0.28395
6	C	-1.60980	1.23697	0.57086
7	H	-1.09085	-2.05963	0.03825
8	H	-3.51026	-1.96294	-0.46840
9	H	-3.49822	2.24528	0.34704
10	H	-1.08561	2.14389	0.85269
11	C	-5.09378	0.24300	-0.38559
12	O	-5.82342	-0.67587	-0.72456
13	H	-5.51454	1.26369	-0.27770
14	C	2.41146	0.76762	-0.52943
15	C	2.65122	-0.75435	-0.20696
16	O	1.36211	-1.17302	0.23934
17	O	1.46261	1.14861	0.46334
18	C	3.67009	-0.95975	0.92763
19	H	3.63881	-2.00605	1.24033
20	H	4.69061	-0.72987	0.60934
21	H	3.42126	-0.34262	1.79045
22	C	3.06209	-1.60861	-1.40602
23	H	4.00538	-1.26021	-1.83659
24	H	3.20223	-2.64479	-1.08755
25	H	2.29941	-1.59738	-2.18428
26	C	1.78389	0.97785	-1.91853
27	H	1.45464	2.01632	-1.99955
28	H	2.49254	0.78052	-2.72729
29	H	0.91259	0.33566	-2.05321
30	C	3.65075	1.65391	-0.40134
31	H	4.43950	1.33742	-1.09011
32	H	3.38912	2.68741	-0.64324
33	H	4.04580	1.63725	0.61392
34	B	0.69368	-0.03456	0.87727
35	F	0.75114	-0.12997	2.33144

Table S4. Atomic coordinates of the **ABPF** anion structure optimized with PBE through CASTEP software.

N°	Atom	x	y	z
1	C	5.42532	5.86128	-9.69095
2	C	5.81772	4.71036	-10.41105
3	C	6.07920	4.76364	-11.77487
4	C	5.97540	5.99280	-12.45754
5	C	5.60304	7.15188	-11.75378
6	C	5.34072	7.08492	-10.38787
7	H	5.89464	3.75600	-9.88475
8	H	6.36192	3.86844	-12.33195
9	H	5.52108	8.10204	-12.28901
10	H	5.04864	7.99068	-9.85150
11	C	6.25572	6.09876	-13.89508
12	O	6.59256	5.18040	-14.63361
13	H	6.13944	7.12704	-14.32030
14	C	6.45720	6.60240	-6.39255
15	C	6.17184	5.04036	-6.17310
16	O	5.71080	4.63896	-7.43850
17	O	5.49012	6.97500	-7.33856
18	C	5.03040	4.84476	-5.12962
19	H	4.72704	3.79056	-5.16553
20	H	5.38764	5.07660	-4.11673
21	H	4.16004	5.46588	-5.37396
22	C	7.37652	4.19748	-5.76783
23	H	7.80504	4.55964	-4.82144
24	H	7.06836	3.15264	-5.61925
25	H	8.15508	4.21500	-6.54037
26	C	7.85940	6.80616	-7.04368
27	H	7.92840	7.85316	-7.36611
28	H	8.65944	6.60456	-6.31883
29	H	7.98048	6.15972	-7.92243
30	C	6.34800	7.47696	-5.14577
31	H	7.05696	7.14168	-4.37380
32	H	6.59064	8.51880	-5.39771
33	H	5.33328	7.45464	-4.73100
34	B	5.01660	5.77656	-8.12174
35	F	3.61968	5.68044	-7.97962

NATURAL ATOMIC ORBITAL AND NATURAL BOND ORBITAL (NBO) ANALYSIS OF THE ATOMS OF THE FORMYL GROUP OF THE **ABP** COMPOUND

Table S5. Occupancy, bond orbital, coefficients and hybrids of the atoms C4, C11, H13 and O12.

**Occupancy**	**Bond orbital**	**coefficients**	**hybrids**				
1.98176	BD(1) C4–C11	0.7255*C (52.64%)	4s	(30.25%)	p2.30 (69.69%)	d0.00	(0.06%)
			-0.0001	0.5500	0.0048	0.0012	-0.0004
			0.8341	0.0066	-0.0025	-0.0030	0.0341
			-0.0036	0.0001	-0.0003	-0.0040	0.0004
			0.0000	0.0000	0.0001	0.0025	0.0000
			-0.0003	-0.0007	0.0010	0.0151	0.0131
			-0.0122	-0.0028			
		0.6882*C (47.36%)	11s	(38.10%)	p1.62 (61.86%)	d0.00	(0.05%)
			0.0003	0.6172	0.0003	0.0055	0.0010
			-0.7824	-0.0014	0.0130	0.0013	-0.0778
			-0.0060	0.0011	0.0004	0.0092	0.0007
			-0.0001	0.0000	0.0052	-0.0005	-0.0006
			0.0001	0.0012	0.0009	0.0185	-0.0050
			-0.0049	.0.0071			
1.99591	BD(1) C11–O12	0.5897*C (34.78%)	11s	(32.18%)	p2.10 (67.71%)	d0.00	(0.11%)
			0.0000	0.5628	-0.0709	-0.0039	-0.0026
			0.5077	-0.0666	0.0010	-0.0001	-0.6380
			0.0432	0.0082	-0.0022	0.0764	-0.0051
			-0.0010	0.0003	-0.0263	0.0067	0.0031
			-0.0008	-0.0041	0.0009	-0.0087	0.0009
			-0.0146	0.0038			
		0.8076*O (65.22%)	12s	(41.15%)	p1.42 (58.36%)	d0.01	(0.49%)
			0.0000	0.6410	-0.0252	0.0012	-0.0002
			-0.4673	0.0050	0.0005	0.0006	0.6000
			-0.0134	0.0014	-0.0011	-0.0709	0.0016
			-0.0002	0.0001	-0.0596	-0.0029	0.0071
			0.0004	-0.0083	-0.0005	-0.0132	-0.0010
			-0.0324	-0.0016			

Table S5. (continued)

Occupancy	Bond orbital	coefficients	hybrids				
1.97832	BD(2) C4–C11	0.5746*C (33.01%)	11s	(0.00%)	p1.00 (99.87%)	d0.00	(0.13%)
			0.0000	-0.0003	0.0000	0.0001	0.0000
			-0.0003	0.0000	0.0000	0.0000	0.1182
			0.0036	0.0031	-0.0011	0.9915	0.0303
			0.0267	-0.0091	0.0025	0.0006	0.0211
			0.0053	-0.0270	-0.0004	0.0032	0.0000
			0.0056	0.0001			
		0.8185*C (66.99%)	12s	(0.00%)	p1.00 (99.65%)	d0.00	(0.35%)
			0.0000	-0.0003	0.0000	0.0000	0.0000
			0.0002	0.0000	0.0000	0.0000	0.1176
			-0.0016	0.0000	-0.0003	0.9912	-0.0134
			0.0002	-0.0030	-0.0043	-0.0001	-0.0365
			-0.0006	0.0448	0.0005	-0.0054	-0.0001
			-0.0093	-0.0001			
1.98639	BD(1) C11–H13	0.7554*C (57.06%)	11s	(30.03%)	p2.33 (69.91%)	d0.00	(0.06%)
			-0.0003	0.5465	0.0412	-0.0026	0.0005
			0.3549	-0.0065	-0.0007	0.0069	0.7512
			0.0245	-0.0117	0.0011	-0.0893	-0.0029
			0.0014	-0.0001	0.0145	-0.0024	-0.0017
			0.0003	-0.0036	-0.0004	-0.0165	0.0022
			-0.0077	-0.0032			
		0.6553*H (42.94%)	13s	(99.94%)	p0.00 (0.06%)		
			0.9996	0.0094	0.0008	-0.0107	-0.0222
			0.0026				

Table S6. Second-order perturbative estimates of "donor-acceptor" (bond-antibond) interactions in the NBO basis of the atoms of the formyl group of the ABP compound.

**Donor NBO (i)**	**Aceptor NBO (j)**	**E(2) (kcal/mol)**	**E(j) – E(i) (a.u.)**	**F(i,j) (a.u.)**
BD(1) C11–O12	BD*(1) C4–C5	1.22	1.63	0.04
BD(1) C11–O12	BD*(1) C4–C11	1.09	1.52	0.04
BD(2) C11–O12	BD*(2) C4–C5	5.06	0.40	0.04
LP(1) O12	BD*(1) C4–C11	1.34	1.15	0.04
LP(1) O12	BD*(1) C11–H13	0.53	1.05	0.02
LP(2) O12	BD*(1) C4–C11	17.96	0.72	0.10
LP(2) O12	BD*(1) C11–H13	23.15	0.62	0.11
